# Prevalence and factors associated with self medication with antibiotics among University students in Moshi Kilimanjaro Tanzania

**DOI:** 10.4314/ahs.v21i2.19

**Published:** 2021-06

**Authors:** Bernard Baltazary Chuwa, Linna Abraham Njau, Kaizali Ivo Msigwa, Elichilia Shao

**Affiliations:** 1 Department of internal medicicine, Kilimanjaro Christian Medical University College Moshi, PO BOX 3010, Moshi Tanzania; 2 Department of Internal Medicine,Kilimanjaro Christian Medical Center, PO BOX 2240, Moshi Tanzania; 3 Clinical Research Unit, Better Human Health Foundation, PO BOX 1348, Moshi Tanzania; 4 MEGA-AFYA and Business Company Limited, PO BOX 6791, Moshi Tanzania

**Keywords:** Self-medication, antibiotics, University students, Moshi, Tanzania

## Abstract

**Background:**

Self medication is a common practice of using medicines without a medical supervision by the people themselves. Self medication is likely to happen when people feel unwell, it is worse in the population with poor helth seeking behavior. Therefore it is important to assess the prevalence and factors associated with self medication with antibiotics among University students in Moshi, Kilimanjaro Tanzania.

**Methods:**

A cross sectional study was conducted from April-August 2019 at two Universities in Moshi, including one medical and one non medical. The study population were undergraduate students aged 18 and above, A self-filled questionnaire was used for data collection and data analyzed using the SPSS version 16 and association was tested using chi square.

**Results:**

Out 374 students enrolled 187 from each University, 126 were female and 248 were male with age ranging from 19 to 35 years with mean age of 23.91 years. The prevalence of self medication with antibiotics was 57% and the most common used antibiotics was amoxicillin with prevalence of 32.08%. The common reported symptoms/diseases were headache (31.02%) followed by malaria and coughing with prevalence of 15.24% and 10.96% respectively. The commonest reasons of self medication reported to be emergency illness (38.77%) and delaying of hospital services (24.33%). The commonest effects reported among respondents which practiced self medication with antibiotics were worsening of the condition that they were suffering in (4.55%) and body rashes (2.67). There was no significant difference between self medication practices among medical and non medical students(p = 0.676)

**Conclusion:**

The prevalence of self medication with antibiotics was high among University students and there is no significant difference in both medical and non medical students. The most feared outcome on self medication with antibiotics is antibiotic drug resistance which leads to treatment failure along with high financial costs and increase mortality rate following microbial infections.

## Introduction

Self medication is a common and regular practice of using medicines without a medical supervision by the people themselves. It is common for people to feel unwell and most of the people have inherited the tendency of using medicines for treating themselves. In this case people tend to use leftover medications at home or buying the medicines from pharmacy following advice from surrounding people like friends and relatives, others tend to use old prescriptions to purchase medicines for current problem that they are having 1.

Self medication with antibiotics has been widely practiced worldwide especially in developing countries including Tanzania. Studies reported the prevalence in developed countries was 3% in northern Europe, 6% in central Europe and 19% in southern Europe in which is more prevalent in the developing countries with loose regulatory system with frequency of SMA ranges from 24% to 73.9% in Africa, 36.1% to 45.8% in middle East 29% in South America and 4% to 75% in Asia^2^. The common reported reasons for self-medication were shortages of drugs at health facilities, long waiting time at health facilities, long distance to health facilities, Inability to pay for health care charges and the freedom to choose the preferred drugs, lack of medical professionals, poor quality of healthcare facilities, unregulated distribution of medicines and patients' misconception about physicians in which were more in developing countries 3.

Self-medication with antibiotics resulting to health problems such as treatment failure, increased incidence of side effects and development of acquired antibiotic resistance that decreases the effectiveness of antibiotics to bacterial infections and this is now becoming a global health problem 4.

Educational interventions on antibiotics uses and limitations targeting community and health facilities, pharmacies and enforcing regulations on non-prescription use of antibiotics, strengthening the antibiotic policies and guide to antimicrobial therapy, establishing a national antibiotic therapeutic advisory committee and establishing a set of national standard treatment guidelines have been recommended 5,6.

Many studies done to assess prevalence and predictors of self medication with antibiotics have been done in general population and medical personnel but published information on University students in Tanzania is limited.

## Methodology

A cross sectional study was done from April-July 2019. The study was conducted at two different Universities in Moshi which included Moshi Cooperative University and Kilimanjaro Christian Medical University College in Moshi Kilimanjaro. Kilimanjaro Region is one of Tanzania's 31 administrative regions. The regional capital is the municipality of Moshi, a sample of 374 undergraduate University students of year 2, 3, 4 and 5 aged 18 years and above from the respective Universities was taken

The researchers reviewed the tools used in similar studies to come out with structured, questionnaire to meet the objectives. Questionnaire was developed according to specific objectives of the study. English language was used in order for all participants to be able to fill them. The tool underwent a pilot phase where 30 students from faculty of nursing who met the inclusion criteria were asked to respond to the questionnaire and the data from the pilot study was not included in this report because the tool had to undergo some corrections and adjustments after piloting

Every day after data collection the questioners were inspected to correct the mistakes and identify the missing data, data obtained were then fed into computer using SPSS version 16 for processing, cleaning and analysis. Association were tested at 95% confidence interval and a significance level of 0.05 (5%). Logistic regression analysis was used to control confounders

Permission letter from Kilimanjaro Christian Medical University Collage and Moshi Co- operative University to undertake the study were obtained. Ethical clearance letter was obtained from the Collage, Informed consent was obtained from each study participant and Confidentiality for data so collected was observed. Information obtained from the study were used for research purposes only, Any participant was free to withdraw from the study if he/she felt uncomfortable in any way and advice and counseling were given to the participants where necessary

## Results

With the response rate of 100%, a total of 374 participants took part in this study with the mean age of 24±2.2 years. This included 187 medical students and 187 non medical students and majority of the participants were male (table 01). Overall prevalence of self medication with antibiotics was found to 57.22%, the prevalence of SMA among medical students was found to be 51% while that of non medical students was found to be 49% (table 02). Age, sex, Marital status, Year of study, University that students belong whether a medical or non medical one and knowledge of the students regarding self medication with antibiotics were associated with self medication with antibiotics but the association was only significant with marital status in which those who are not in union had less odds of practicing SMA than their counter part (table 03). The most common symptoms/diseases that lead students to practice self medication with antibiotics were headache which contributed about 31% of all cases and least being other symptoms apart from those mentioned in the table, these include sore throat, tonsillitis, typhoid, peptic ulcers etc contributing about 0.3% each (table 04). Antibiotics used by students for self medication include amoxicillin which was the most commonly used in 32.08% 0f the cases and ceftriaxone being the least with prevalence of 0.5% (table 05). Other antibiotics which were also used and are not mentioned in the table include penicillin v in 0.3% of the cases. Majority of the students obtained information about these antibiotics from the pharmacies in 56.95% while radio and television being the least sources of information (table 06). The frequently reported reasons for self medications with antibiotics are shown in the table 07 with the leading reason being emergency illness (38.77%). The commonest effects reported among respondents which practiced self medication with antibiotics were worsening of the condition that they had (4.55%) and body rashes (2.67%) table 08.

**Table 01:** Socio-demographic characteristics of participants (N=374)

Variables	Frequency	percentage
**Sex**		
Male	248	66.3
Female	126	33.7
**Age**		
19–24	269	71.9
25–30	103	27.5
31–35	2	0.5
**University**		
KCMUCo	187	50
MoCU	187	50
**Marital status**		
Single	333	89
Married	24	6.4
Divorced	4	1.1
Cohabiting	13	3.5
**Year of study**		
2	105	28.1
3	236	63.1
4	14	3.7
5	19	5.1

**Table 02:** Prevalence of self-medication with antibiotics (N=374)

Variables	Frequency	Percentage
Have you ever self medicate with antibiotics within the past one year?		
Yes	214	57.22
No	157	41.99
I don't remember	3	0.8

**Table 03:** Factors associated with self-medication (N=374)

Variables	frequency	percentage	OR	95% CI	P-value
Age					
21–28	360	96.3	0.9	0.34–2.93	0.9
29–36	14	3.7			
Sex					
male	248	66.3	0.7	0.46–1.11	0.128
female	126	33.7			
University					
KCMUCo	187	50	0.92	0.608–1.38	0.676
MoCU	187	50			
knowledge on self-medication with antibiotics					
yes	301	80.5	0.651	0.29–1.43	0.431
no	73	19.5			

**Table 04:** Common symptoms/diseases treated by self-medication with antibiotics (N=331)

Variables	Frequency	Percentage
Headache	116	31.02
Diarrhoea	21	5.61
Malaria	57	15.24
Fever	39	10.43
Coughing	41	10.96
Eye infection	8	2.14
Skin infection	14	3.74
Injury	11	2.94
Others	2	0.53

**Table 05:** Common antibiotics used during self-medication (N=226)

Variables	Frequency	Percentage
Amoxicillin	120	32.08
Doxycycline	16	4.29
Tetracycline	7	1.87
Erythromycin	4	1.07
Chloramphenicol	4	1.07
Metronidazole	33	8.82
Ciprofloxacin	8	2.14
Ampiclox	20	5.35
Ceftriaxone	2	0.53
Others	12	3.21

**Table 06:** Sources of information about antibiotics used for self medication (N=353)

Variables	Frequency	Percentage
Pharmacies	233	56.95
Government health institution	80	21.4
Friends/Relatives	6	1.6
Information from internet	10	2.67
Read from newspaper	3	0.8
Radio/Television	2	0.53
Others	19	5.08

**Table 07:** Reasons for self medication with antibiotics (N=317)

Variables	Frequency	Percentage
Emergency illness	145	38.77
Distance to the health facility	36	9.23
Proximity of the pharmacy to home	19	5.08
Health facility charges	19	5.08
No medicine in health facility	4	1.07
Delaying of the hospital services	91	24.33

**Table 08:** Effects observed after self-medication with antibiotics (N=49)

Variables	Frequency	Percentage
Body rashes	10	2.67
Swollen face	7	1.87
Yellowish eyes	5	1.34
Vomiting	1	0.27
Diarrhea	3	0.8
Condition worsened	17	4.55
Others	6	1.6

## Discussion

The results shows the over roll prevalence of self medication with antibiotics among University students was 57.22% and the most common used antibiotics was amoxicillin with prevalence of 32.08%. Majority of the students obtained information about antibiotics from the pharmacies in 56.95% while other obtained the information from radio and television. The common reported symptoms/diseases were headache (31.02%) followed by malaria and coughing with prevalence of 15.24% and 10.96% respectively. The commonest reasons of self medication reported to be emergency illness (38.77%) and delaying of hospital services (24.33%). The commonest effects reported among respondents which practiced self medication with antibiotics were worsening of the condition that they were suffering from (4.55%) for example if someone had headache instead of improving after self medication with antibiotics the headache got worsened followed by body rashes (2.67%).

In this study, the prevalence of self medication with antibiotics among University students was 57.22%. This prevalence is slightly lower than that observed in study done in Siha district (58%) in Tanzania^2^. The difference is low between these two populations that shows the issue might be more than education may be attitude of the people toward self medication and prevalence is lower than Karachi, Pakistan (76%) while the study population was the same with ours, the observed difference may be due to economic differences between the countries, Pakistan might have high access rate to antibiotics compared to Tanzania8 but higher than that observed in study done in Mbeya, Tanzania (19.7%), South Ethiopia (27%), Nigeria (38.8%), Alexandria, Egypt (53.9%) and Sourthen China (47.8%)^2^,^9^–^11^. According to this percentage (57.22%) of students had self medicated with antibiotics without clinical proper advice and the most feared issue associated with this practice is antibiotic resistance, so education on effects of self medication with antibiotics is highly required in general population as in educated population the prevalence is this high. So this study brings out this issue of relation between self medication and antibiotics resistance in Tanzania could be a useful topic for future studies whereby they can look for factors other than education.

In this study, emergency illness and delaying hospital services were the commonest reported factors associated with self medication with antibiotics unlike other studies done in Kilimanjaro where poor knowledge concerning how to use antibiotics, high charges, emergency illness and availability of pharmacies which facilitated treatment without seeing a doctor were the factors led to self medication with antibiotics^7^,^12^. In Sudan, pharmacies have low cost compared to the other health facilities as they charge consultation and laboratory fees this contributed more in self medication with antibiotics, in China, advanced age and high allowance were risk factors for self medications with antibiotics^2^,^13^. Unlike the ideal setting, our findings were different from other studies because our participants belong to a well educated category, located in area with available health services such as pharmacies, dispensaries and hospital and most of the health charges are covered by health insurance companies including NHIF but the delaying of hospital services which is the reason for self medication with antibiotics so we think health system has challenge on doctor to patient ratio which need to be addressed.

It was surprising to find that there was no significant difference in prevalence rates of self medication among medical students (51%) and non medical students (49%) with P value of 0.676. This finding is correlating with the study done in Karachi, Pankistan among medical and non medical University students^8^. We were thinking that medical students would have low prevalence of self medication with antibiotics because they have more knowledge about antibiotics. It is difficult to know the reason for this finding from the data we collected but we think the proximity and interaction for these two Universities might have an influence on this finding because non medical students may tend to seek medical advice from medical students knowing that they have the knowledge and this becomes the interesting subject for future research in other population.

In this study, we found that the common reported reasons for using medication without prescription instead of going to health facilities among medical University students were delaying of hospital services (35.3%) and emergency illness (34.2%) and in non medical students the commonest reason was emergency illness (43.3%). The sources of medicines to both medical and non medical students were found to be pharmacies with prevalence of 70.6% in medical students and 43.3% in non medical students we think medical students get medicines in pharmacy with higher prevalence compared to other sources because the University is surrounded with high number of pharmacies. Amoxicillin and metronidazole were the most common reported drugs for self medication in medical students 32.6% and 10.7% respectively and in non medical students 31.6% and 7% respectively and ampiclox was also the common drug in non medical students with prevalence of 6.4%, in Moshi municipal, Kilimanjaro ampiclox (27%) and amoxicillin (18.4%), Amoxicillin was the most frequently used among study done in Siha, Kilimanajaro (43%), Trujilo (20.33%) and Karachi (41.4%) and in Punjani, Pakistan the most frequently used was metronidazole^7^,^12^,^14^–^15^. So in all these studies, amoxicillin was the commonest antibiotics used during self medication with antibiotics this might be due to its availability and nature of the conditions such as coughing.

## Strengths and limitations

According to our knowledge this is probably the first study to study the difference in self medication with antibiotics between medical and non medical University students in Tanzania and Africa. Our study was done on well described population with similar characteristics such as all students' health charges are covered by insurance companies, all are University students and they are all surrounded by health facilities.

Majority of non medical participants had difficult to understand because of language barrier due to technical names so we explained the parts they faced difficulty. Some of participants had difficult to recall the names of antibiotics and ended up mentioning pain killers such as paracetamol and ibuprofen and anti protozoa such as ALU in others options, so we considered these participants not to have self medicate with antibiotics therefore this may affect the true value of the prevalence of self medication with antibiotics. There were limited published studies which compare self medication with antibiotics between medical and non medical University students especially in Tanzania and Africa.

## Conclusion

From our study findings self medication with antibiotics has high prevalence among University students and there is no significant difference in both medical and non medical students. The most feared outcome on self medication with antibiotics is antibiotic drug resistance which leads to treatment failure along with high financial costs and increase mortality rate following resistant microbial infections. Our study has opened a way for further research in this issue to look for the changing trends of self medication with antibiotics so as to plan in controlling antibiotic related health risks, besides showing that it is a real problem and should not be ignored

## Recommendations

Health education should be promoted and provided to University students with regards to the adverse effects of practicing self medication with antibiotics. Ministry of health should strengthen supportive supervision to health facilities and pharmacies to ensure those health facilities strictly follow the rule of dispensing antibiotics. Ministry of health should improve the quality of health care services in public health centers to increase customer satisfaction by recruiting more medical personnel so as to reduce the burden of patients and solve the problem of delaying of services.

## Comflict of interests

None declared.
